# Real-Time Dynamic Path Planning of Mobile Robots: A Novel Hybrid Heuristic Optimization Algorithm

**DOI:** 10.3390/s20010188

**Published:** 2019-12-28

**Authors:** Qing Wu, Zeyu Chen, Lei Wang, Hao Lin, Zijing Jiang, Shuai Li, Dechao Chen

**Affiliations:** 1 School of Computer Science and Technology, Hangzhou Dianzi University, Hangzhou 310018, China; wuqing@hdu.edu.cn (Q.W.); zeno_chen@163.com (Z.C.); 181050059@hdu.edu.cn (L.W.); 171050026@hdu.edu.cn (H.L.); jzj@hdu.edu.cn (Z.J.); 2 Department of Computing, The Hong Kong Polytechnic University, Hung Hom, Kowloon, Hong Kong 999077, China; shuaili@polyu.edu.hk

**Keywords:** hybrid optimization algorithm, mobile robot, real-time path planning, dynamic obstacle avoidance, beetle antennae search algorithm (BAS)

## Abstract

Mobile robots are becoming more and more widely used in industry and life, so the navigation of robots in dynamic environments has become an urgent problem to be solved. Dynamic path planning has, therefore, received more attention. This paper proposes a real-time dynamic path planning method for mobile robots that can avoid both static and dynamic obstacles. The proposed intelligent optimization method can not only get a better path but also has outstanding advantages in planning time. The algorithm used in the proposed method is a hybrid algorithm based on the beetle antennae search (BAS) algorithm and the artificial potential field (APF) algorithm, termed the BAS-APF method. By establishing a potential field, the convergence speed is accelerated, and the defect that the APF is easily trapped in the local minimum value is also avoided. At the same time, by setting a security scope to make the path closer to the available path in the real environment, the effectiveness and superiority of the proposed method are verified through simulative results.

## 1. Introduction

In recent decades, path planning has had important applications in many areas, such as mobile robots [1,2,3,4,5,6], unmanned aerial vehicle (UVA) [7,8], game artificial intelligence (AI) automatic pathfinding [9], etc. [10,11,12,13]. In particular, mobile robots have been put into practical use in many industries. For example, [14] introduced the practical application of sweeping robots for daily household cleaning, [15] proposed a robotic automatic battery sorting system for improving battery sorting efficiency, and [16] introduced many applications of mobile robots in agriculture: tilling, seeding, harvesting, etc.

For mobile robots, path planning consists of finding a feasible path to the target point in the workspace [17,18,19,20,21,22,23]. There are many related research results for this research direction [24,25,26,27,28]. For example, some traditional methods are: artificial potential field (APF), probabilistic roadmap method (PRM), and rapidly-exploring random trees (RRT) [29,30,31]. The paper presented in [32] used a hybrid algorithm of A* and RRT for indoor navigation of unmanned aerial vehicle. In [33], they proposed a bidirectional RRT based on potentially guidance to quickly plan paths in a messy environment. Most of these methods have certain advantages in terms of speed, but the path obtained lacks optimization. Some heuristic algorithms, such as A*, D*, are widely used in industry [34,35,36]. Xin et al. [37] proposed an improved A* algorithm, which extends the neighborhood propagation of the standard A* algorithm. In [38], the modified A* algorithm is used to plan the path of the mobile robot. The connection between the path points of the A* algorithm is mainly modified. But these methods are mainly used in globally-known environments. There are also some intelligent optimization algorithms, such as ACO algorithm, genetic algorithm, and PSO algorithm, which also has corresponding results [39,40,41,42]. In [43], by combining artificial bee colony algorithm and evolutionary programming algorithm, they proposed a new path planning algorithm applied to path planning in two-dimensional static environment. In [44], they designed a non-dominated sorting genetic algorithm for multi-objective path planning in static environments. In [45], a heuristic PSO algorithm is proposed, which improves the PSO planning deficiency to a certain extent but only verifies the effectiveness of the algorithm in static environment. However, due to the characteristics of the group intelligence algorithm, the heuristic PSO algorithm is not particularly ideal in terms of time.

As can be seen from the above introduction, it is still necessary to study real-time dynamic path planning. It can also be seen that the research trend of path planning in recent years is also realized by a mixture of various algorithms to obtain better results.

The structure of this paper is organized as follows. Section 2 includes the definitions and formula descriptions for real-time path planning problems. In Section 3, we introduce the design ideas and implementation steps of the proposed beetle antennae search (BAS)-APF method in stages. Demonstration of the effectiveness of the algorithm through some numerical simulations for maps with dynamic obstacles is shown in Section 4. Finally, the conclusions are presented in Section 5. The main contributions of this paper are listed below.

- A novel hybrid intelligent optimization method, named BAS-APF, is proposed and applied to mobile robot dynamic path planning. The proposed method is divided into two phases. First, a feasible path from the current point to the end point is initialized based on the proposed algorithm. Then, path tracking is performed, and the path is optimized during path tracking without affecting the real-time performance of the algorithm.
- The proposed method has a good performance in both the planning time and the path length. And the advantages in planning speed are outstanding.
- The proposed method is simulative in our pre-set simulation environment maps and the real map collected by sensors, and its effectiveness and superiority are verified by comparison with other algorithms.

## 2. Problem Formulation

This section describes the definition of the path planning problem discussed in this article, some of the symbols used, and a brief introduction to the design basis of the proposed method. The method we proposed is called the BAS-APF. Therefore, as a background, as well as a brief introduction to the BAS algorithm and the APF algorithm, given below.

BAS is a relatively novel intelligent optimization algorithm. The design idea is inspired by the foraging behavior of beetles in nature. By optimizing the beetle’s foraging process and the concept of pheromone, an optimization algorithm with faster optimization is proposed [46].

APF is a classic obstacle avoidance method. The basic idea is to abstract the motion of the robot in the surrounding environment into the motion in the artificial potential field and guide the robot movement through the force of the potential field. But this method easily falls into local extremum.

The above is a brief introduction to the basic algorithm of the proposed method. The following is a brief introduction and definition of the problem to be solved in this paper. The specific discussion in this paper is the real-time path planning problem in dynamic environment. For this problem, we need to plan a safe path from the start point to the end point as quickly as possible. The next points are taken into consideration for the hybrid BAS-APF path planning method.

- $x_{\mathrm{sta}}$ and $x_{\mathrm{tar}}$ represents the start and target points of the plan, respectively.
- Express the configuration space in this paper as a set of $Z\subset R^{n},n\in N$ and n≥2, where *n* is the dimension of the configuration space. $Z_{\mathrm{obs}}\subset Z$ is a set of obstacle areas in our configuration space. $Z_{\mathrm{fre}}\subset Z$ is a set of passable areas in our configuration space that can be obtained according to Formula (1). Another thing to note is that the dynamic obstacles used in this paper, the value of *Z* will change in real time:
  (1) $$Z_{\mathrm{fre}}=Z\setminus Z_{\mathrm{obs}}.$$
- The path obtained by the planning defined as P is a set of points $\left\{p_{1},p_{2},p_{3}\ldots p_{\max}\right\}$, where $p_{i}$ is the *i*-th path point.
- Path planning can be defined as: setting the function to be optimized f(·) of the sampling point, and let $f\left(\cdot \right)\to 0^{+}$ during the planning process.
- The path optimization process can be defined as: setting the cost function g(·) of the path and obtaining min.g(·) during each optimization process.

In general, we evaluate the path based on some criteria, such as path length, planning time, etc. Therefore, we only need to minimize g(·), while ensuring the planned time *t*. In addition, this article is mainly focused on two-dimensional environment, but this method is applicable to the case of higher dimensions.

## 3. Methodology

**Figure 1:**
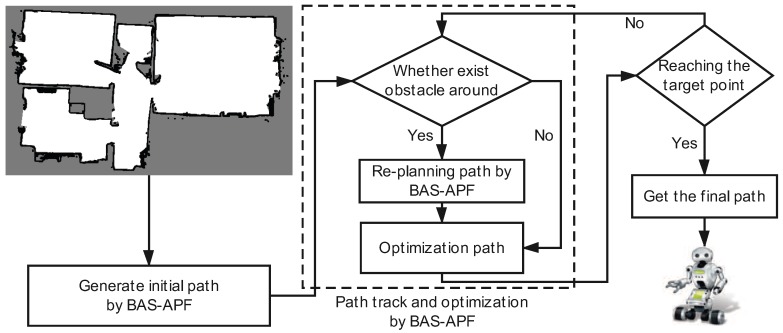
The schematic diagram of the proposed beetle antennae search (BAS)-artificial potential field (APF) method applied to mobile robot path planning in both static and dynamic environments.

In this paper, we proposed a novel hybrid BAS-APF method for real-time path planning. This method can be applied to mobile robot path planning in both static and dynamic environments. The entire method flow is shown in Figure 1.

The proposed method is used to generate a safe path from a preset starting point to a target point. Our approach aims to solve the real-time path planning problem of mobile robots in dynamic environments. The map information of the environment needs to be processed after being collected from the sensor. Some of the map files used in this paper are in our own preset simulation map, and the other part is obtained after the real environment is collected and processed by the sensor. Below, we have a detailed introduction to the proposed method.

### 3.1. Proposed BAS-APF Method

Path planning in a dynamic environment places high demands on the real-time and security of the planning algorithm. For these two requirements, we first avoid collisions by setting up a layered detector and a well-designed cost function. At the same time, in order to meet the requirements of real-time, the proposed method first gives the initial path, and then gradually optimizes and updates the path during the tracking process of the mobile robot.

The proposed method can be divided into two phases as a whole. The first phase is to generate the initial path, and the second phase is to trace the trajectory of the initial path and optimize the iterative update.

#### 3.1.1. Initial Path Generation

First, according to the pre-setting starting point $x_{\mathrm{sta}}$, the detection range τ, the direction vector d and the Formulas (2) and (3), select two candidate points $x_{l},x_{r}$ within the perimeter detection range. $x_{\mathrm{sta}}$ is the initial position of the longicorn in BAS algorithm. d is the direction that beetle will expore.

(2) $$d=\frac{\mathrm{rands}\left(k,1\right)}{{∥\mathrm{rands}\left(k,1\right)∥}_{2}},$$

where rands(·) is a random function, each time a *k*-dimensional direction vector d is randomly selected, and *k* is the dimension of the space to be planned.

(3) $$\begin{array}{c} x_{l}=x+d\tau \eta^{i-1},\\ x_{r}=x-d\tau \eta^{i-1}, \end{array}$$

where $x_{l}$ and $x_{r}$ are the coordinates of the candidate points. τ is the size of the detection range, and η is the attenuation rate of the detection range. As the number of iterations increases, the detection range of the agent will gradually shrink, which can also reduce the possibility of falling into local extremum to some extent.

Then, according to Formula (12), the cost function of current point, we calculate $f(x_{l})$ and $f(x_{r})$ to select a better candidate point, respectively. And according to the Formula (4) and (5), the mobile robot moves one step in the direction of the better point. $x_{\mathrm{nex}}$ is the position that the robot will move next, and its formula is as follows:

(4) $$x_{\mathrm{nex}}=x+s\chi^{n}\mathrm{sign}\left(f\left(x_{l}\right)-f\left(x_{r}\right)\right)d,$$

where *s* represents the current step size, χ represents the step decay rate, and sign(·) is a symbolic function that extracts the sign of a real number.

(5) $$\mathrm{sign}\left(x\right)=\left\{\begin{array}{cc} 1,\; & x>0,\\ 0,\; & x=0,\\ -1,\; & x<0. \end{array}\right.$$

Add the coordinates to the alternate path after getting $x_{\mathrm{nex}}$. The candidate path is evaluated according to the evaluation Formula (6). If the evaluation value is better, the current path is replaced; otherwise, the candidate point is re-selected. Iterate through the above process until the target point is reached:

(6) $$g\left(p\right)=\sum_{i=1}^{N}{\left(p_{i}-p_{i-1}\right)}^{2},$$

where *N* is the number of path points, and *i* is the *i*-th path point. The above steps can already achieve path planning from the start point to the end point, but it may fall into local extremes, resulting in slow planning. Therefore, we speed up planning by designing appropriate cost functions and introducing artificial potential fields. A spline curve is also introduced to fit the path to achieve a smooth path effect.

#### 3.1.2. Add Artificial Potential Field Function

**Figure 2:**
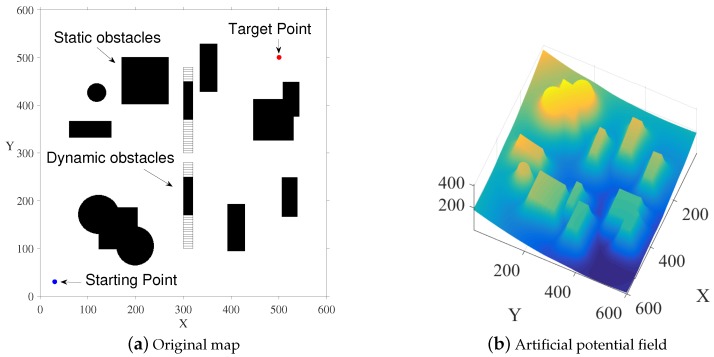
Original map and artificial potential field (APF) visualization.

Artificial potential field (APF) is a trajectory planning method based on artificial space field. The basic idea is as follows: First, the robot is regarded as a point in the space to be planned, and the artificial potential field fills the entire space to be planned. The construction method of the artificial potential field can cause the robot to be attracted to the target point away from the obstacle space. If the potential field is constructed reasonably, the artificial potential field will reach a global minimum at the end point. However, it is difficult to construct such a potential field, and even if such a potential field is constructed, it is not common to all environments. The implementation of the entire artificial potential field can be seen more intuitively through Figure 2.

Next, we will explain the construction methods of the gravitational field and the repulsive field in the artificial potential field. The gravitational field needs to satisfy the increase in the distance between the current point and the target point, so the easiest way is to set a function that increases linearly with distance. However, this is a defect in the potential field; that is, when the current point is too far from the target point, the gravity is too large. Therefore, an additional quadratic form function is set, and the threshold is set to counteract the effect of the linearly increasing gravitational pull. The specific gravitational field Formula (7) is as follows.

(7) $$U_{\mathrm{att}}=\left\{\begin{array}{cc} \frac{1}{2}\epsilon d_{g}^{2}, & \;\mathrm{if}\;d_{g}<d_{\mathrm{gra}},\\ \epsilon d_{\mathrm{gra}}d_{g}-\frac{1}{2}\epsilon d_{\mathrm{gra}}^{2}, & \;\mathrm{if}\;d_{g}>d_{\mathrm{gra}}, \end{array}\right.$$

where ϵ is the gravitational factor that determines the gravitational pull of the current point, and $d_{g}$ is the distance from the current point to the target point. $d_{\mathrm{gra}}$ is the distance threshold of the gravitational field. The calculation of gravitation is to obtain the gradient information of the gravitational field and take the negative gradient. See Formula (8) for specific gravity settings,

(8) $$F_{\mathrm{att}}=\left\{\begin{array}{cc} -\epsilon d_{g}, & \;\mathrm{if}\;d_{g}<d_{\mathrm{gra}},\\ -\epsilon d_{\mathrm{gra}}+\frac{\epsilon d_{\mathrm{gra}}}{d_{g}}, & \;\mathrm{if}\;d_{g}>d_{\mathrm{gra}}. \end{array}\right.$$

Then, we need to build a repulsive field. The repulsive field needs to ensure that the farther away from the obstacle, the smaller the repulsive force, and the distance threshold $d_{\mathrm{rep}}$ is set in order to prevent repelling interference from distant obstacles. When the current point distance obstacle is greater than the threshold, the obstacle does not generate a repulsive force to the current point. The specific repulsive field structure is shown in Formula (9).

(9) $$U_{\mathrm{rep}}=\left\{\begin{array}{cc} \frac{1}{2}\mu {\left(\frac{1}{d_{r}}-\frac{1}{d_{\mathrm{rep}}}\right)}^{2}, & \;\mathrm{if}\;d_{r}<d_{\mathrm{rep}},\\ 0, & \;\mathrm{if}\;d_{r}>d_{\mathrm{rep}}, \end{array}\right.$$

where μ is the repulsion factor, $d_{r}$ is the distance from the current point to the obstacle, and $d_{\mathrm{rep}}$ is the distance threshold for the repulsion. Similarly, we can get a specific repulsion value by grading the repulsion field. The specific form is as in Formula (10),

(10) $$F_{\mathrm{rep}}=\left\{\begin{array}{cc} \mu {\left(\frac{1}{d_{r}}-\frac{1}{d_{\mathrm{rep}}}\right)}^{2}\frac{1}{d_{r}^{2}}, & \;\mathrm{if}\;d_{r}<d_{\mathrm{rep}},\\ 0, & \;\mathrm{if}\;d_{r}>d_{\mathrm{rep}}. \end{array}\right.$$

Finally, according to the Formula (11), we combined it with attraction and repulsive force to obtain a complete force to guide the robot to the target point.

(11) $$F=F_{\mathrm{att}}+F_{\mathrm{rep}}.$$

#### 3.1.3. Design Cost Function

**Figure 3:**
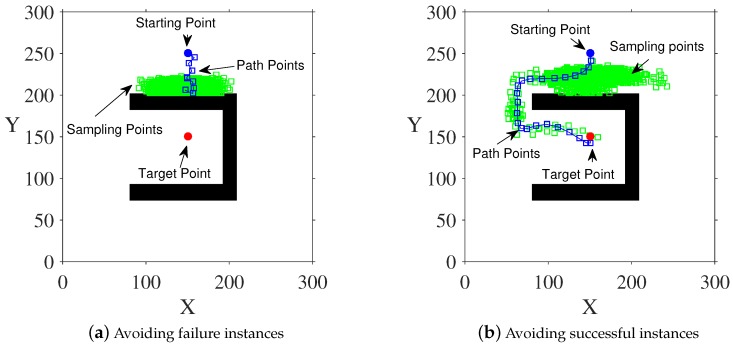
Avoid instances of local extremum.

In the path planning process, due to the scope of the sensor, there may be cases where the agent is trapped at a local minimum and cannot reach the target point. This situation is called a local extremum problem. As shown in Figure 3a, in this example, the robot is stuck at a local minimum and cannot reach the target point. We avoid this problem by designing a suitable cost function. By layering the scope of the robot’s detection, we add the corresponding penalty to the cost function according to the location of the obstacle, which helps to escape the local minimum. See Figure 3 for specific effects.

The green squares in Figure 3 represent sampling points, and the blue squares represent path points. Figure 3a is an example of local extremum avoidance when the detection range of the agent is not layered, and the agent is more likely to fall into local extremum. In Figure 3b, we layer the detection range of the agent. Comparing the two sub-figures, it is found that layering the detection range is beneficial to improve the local extremum avoidance ability of the proposed method.

The specific cost function is shown in Formula (12). We divide the detection range of the agent into two layers. When the agent detects obstacles O in the internal detection range, it adds a penalty factor α to the cost function. When the outer layer of the detection range has an obstacle, the reward coefficient β is added to the cost function. When there is no obstacle within the detection range, the resultant force of the potential field is directly used as the cost function value.

(12) $$f\left(x\right)=\left\{\begin{array}{cc} \alpha {\left(x-x_{\mathrm{tar}}\right)}^{2}+F\left(x\right), & \;\mathrm{if}\;O\in D_{\mathrm{in}},\\ \beta {\left(x-x_{\mathrm{tar}}\right)}^{2}+F\left(x\right), & \;\mathrm{if}\;O\in D,\\ F(x), & \;\mathrm{if}\;O\notin D, \end{array}\right.$$

where α,β is the reward coefficient and penalty factor, F(x) is the potential field force of the current point, $D_{\mathrm{in}}$ is the inner detection range, and *D* is the detection range.

#### 3.1.4. Spline Curve

The initial path we get may be too tortuous. Therefore, we have adopted a more common curve fitting method: B-spline curve [47]. The B-spline curve can achieve the effect of modifying the local path without changing the shape of the entire path [48,49,50]. $N_{i,p}$ is a normalized B-spline basis function defined by the following Cox-deBoor recursive Formula (13),(14).

(13) $$N_{i,0}\left(u\right)=\left\{\begin{array}{cc} 1, & \;\mathrm{if}\;u_{i+1}\leq u<u_{i+1},\\ 0, & \;\mathrm{if}\;;otherwise, \end{array}\right.$$

where $N_{i,0}\left(\cdot \right)$ is piecewise constant 1 or zero.

(14) $$N_{i,p}\left(u\right)=\frac{u-u_{i}}{u_{i+p}-u_{i}}N_{i,p-1}\left(u\right)+\frac{u_{i+p+1}-u}{u_{i+p+1}-u_{i}}N_{i+1,p-1}\left(u\right),$$

where $N_{i,p}\left(\cdot \right)$ is the *i*-th B-spline basis function of degree *p*. The $u_{i}$ represents the *i*-th item in a set of non-decreasing numbers $\left[u_{0},u_{1},\ldots ,u_{\max}\right]$.

In summary, the entire path planning process is now complete, and the pseudo code that generates the initial path can be viewed in Algorithm 1. It is worth noting that we input the map environment information to simulate the overall simulation environment, but the global environment information is not used in the planning process. That is to say, in every step of path planning, the whole environment information is not used; only the environment information around the robot is used. The content of the next subsection is the tracking optimization process for the initial path.
**Algorithm 1:** BAS-APF design process 
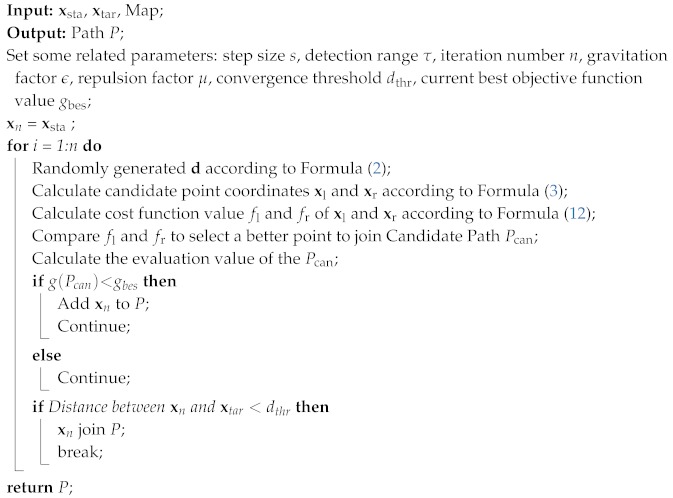


### 3.2. Real-Time Path Tracking

The whole process of path tracking is roughly as shown in Algorithm 2. First, in order to speed up planning and reduce the amount of computation, the proposed method does not re-plan the path at each step of the tracking but, instead, constructs the detector during the tracking process. The detector has a radius that is twice the step size. Algorithm 1 is called to re-plan the subsequent path only if there is an obstacle in the probe’s detection range. At the same time, we also set limits on the number of re-planned algorithm iterations, so although the obtained path may not be globally optimal, it guarantees the real-time performance of the algorithm.
**Algorithm 2:** Real-time path tracking 
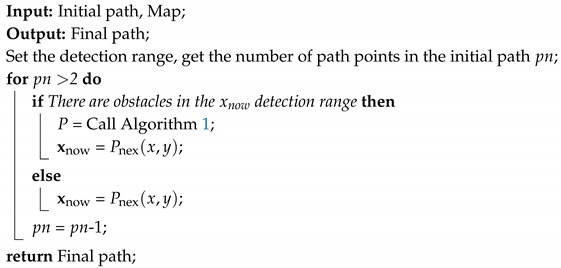


The above is the complete process of the proposed method.

## 4. Simulations

In order to verify the effectiveness of the proposed method, we performed a series of simulations in MATLAB. In this section, we selected a few representative maps to visualize the results: one is our pre-set simulation map. The other two were obtained by modeling the real room environment. The length of the following maps is in centimeters. At the same time, comparison simulations with several other algorithms were also carried out. By comparing the simulative results, the superiority of the algorithm is verified. In addition, we also made a visual process diagram of the pathfinding process of the BAS-APF method, which can visually see the entire path optimization process.

### 4.1. Simulation Map Results Using Virtual Map

**Figure 4:**
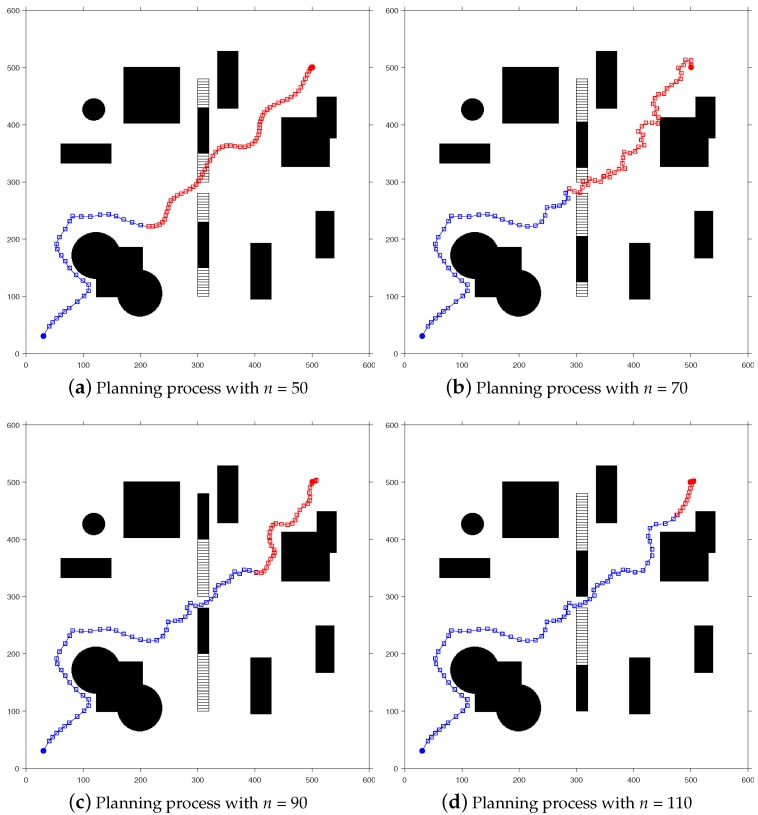
The process of exploring a path on a simulative map with dynamic obstacles.

This section mainly shows the simulation results of the proposed method on the simulated map and the visualization of the planning process. In addition, a comparison of the selection criteria of the comparison algorithm and the results of the single path planning is also introduced. The preset simulation map (Map 1) resolution is 600 × 600, and the map contains multiple static obstacles and two regularly moving dynamic obstacles. Taking Figure 4 as an example, in an environment of 600 cm × 600 cm, dynamic obstacles move 5 cm in each step of the robot, with 130 steps in total, i.e., 650 cm in total, with a total planning time of 0.287 s. The obstacles move with the velocity being 22.65 m/s, and the white hollow square is the trajectory of the obstacles.

A visual representation of the proposed method on Map 1 is shown in Figure 4, where the blue open squares indicate the determined path points, and the red open squares indicate the path points to be determined. The black area represents the obstacle, and the white hollow square represents the movement trajectory of the dynamic obstacle. Each square represents one step of the robot’s movement, and *n* represents the number of iteration steps.

Regarding the choice of comparison algorithm, we make a decision based on the following considerations. Firstly, because our proposed method refers to the idea of APF algorithm, in order to highlight the superiority of hybrid algorithm, we choose APF as one of the comparison algorithms. In addition, since the BAS algorithm is an intelligent optimization algorithm, we chose a classic intelligent optimization algorithm: ACO algorithm as a comparison algorithm. Based on the comprehensive consideration of the comparison algorithm, we also selected a sampling-based algorithm: RRT. The superiority of the BAS-APF method is verified by comparison with multiple different types of comparison algorithms.

**Figure 5:**
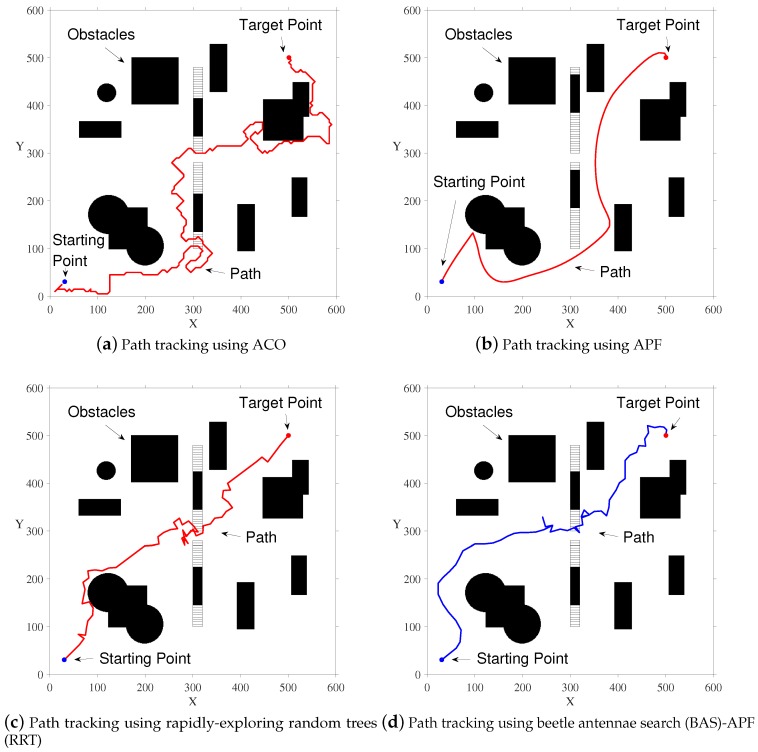
Simulation results of simulation map.

In Figure 5, we show the results of four different contrast algorithms on a simulated map. It is worth mentioning that the number of iterations of ACO is 1000, and the number of ants is 50. The blue dot represents the starting point and the red dot represents the ending point. By comparing the four subgraphs, we find that the final path obtained by our proposed algorithm is smoother and shorter, and the final path is not too close to the obstacle, and the safety factor is higher.

### 4.2. Simulated Results Using Real Map

Considering the practical applicability of the algorithm, in this section, we have selected two processed actual maps for simulation. Since this paper mainly verifies the obstacle avoidance ability of the proposed method in the dynamic environment, we added two dynamic obstacles that move regularly along the horizontal direction in the first real map (Map 2). And the size of Map 2 is 637 × 355. Similarly, the second real map (Map 3) adds two dynamic obstacles that move regularly in the vertical direction. The specification of Map 3 is 597 × 375.

**Figure 6:**
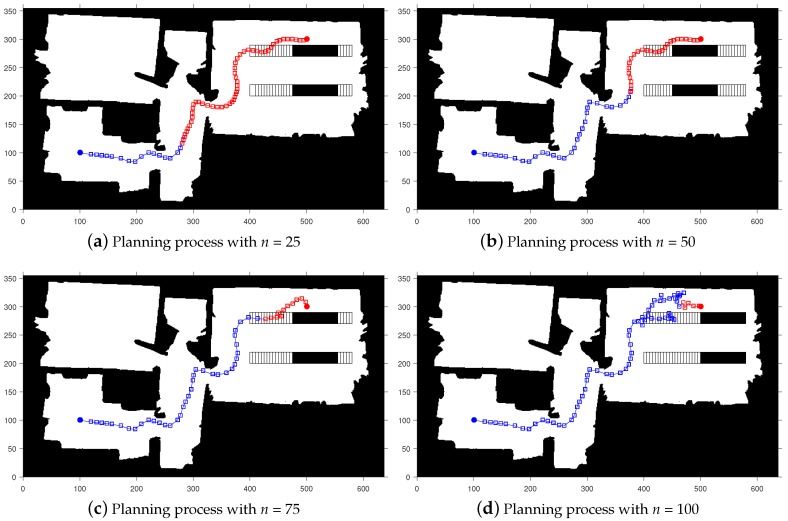
The process of exploring a path on the map with horizontally dynamic obstacles.

Figure 6 shows the planning process of our algorithm on Map 2. At the same time, in order to better reflect the entire tracking process, we select the process screenshot of the re-planning. It can be seen that, since in Figure 6a,b, there are no obstacles in the surrounding area. In order to improve the tracking speed, no re-planning is performed. In Figure 6a,d, there are dynamic obstacles in the peripheral area where collisions may occur. The algorithm re-plans and successfully avoids obstacles in reaching the end point.

**Figure 7:**
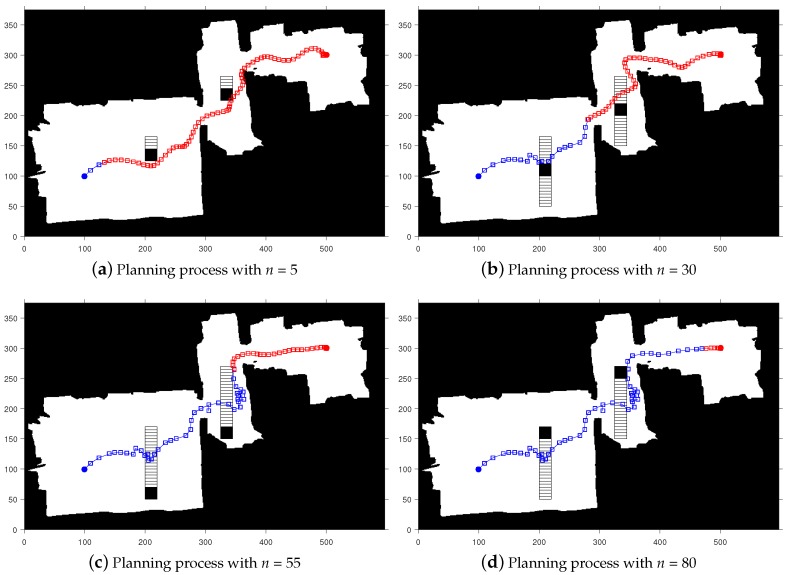
The process of exploring a path on a map with vertical dynamic obstacles.

Similarly, Figure 7 shows the tracking process on Map 3 in its entirety. It can be seen from the two figures that the algorithm has good adaptability in different environments and can plan a safe path, while dynamically avoiding obstacles.

**Figure 8:**
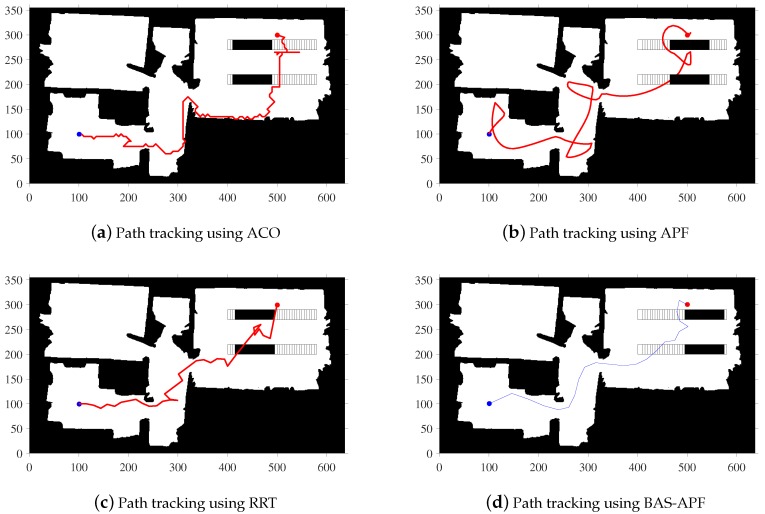
Comparative simulation results on a map with horizontal dynamic obstacles.

In order to more intuitively reflect the superiority of BAS-APF algorithm, we also conducted a comparison simulation on Map 2 and Map 3. Figure 8 shows the tracking results of the BAS-APF and comparison algorithm in Map 2. As can be seen from the figure, the path obtained by ACO algorithm is too close to the obstacle, and the path obtained by APF is smooth, but there are too many useless turns. The path obtained by RRT algorithm is too tortuous, and there is also a case where a useless return path is taken. The path obtained by BAS-APF takes into account the balance between path length and path smoothness.

**Figure 9:**
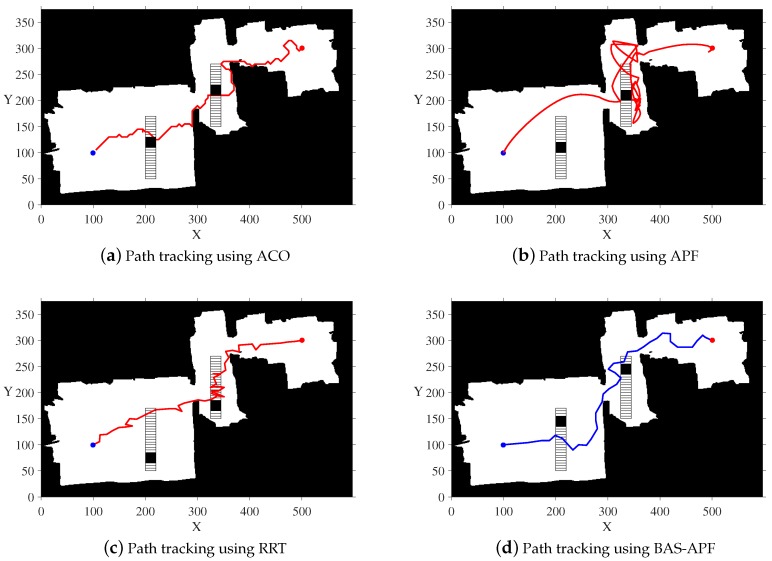
Comparative simulation results on a map with vertical dynamic obstacles.

Similarly, in Figure 9, we show the results of planning for multiple planning algorithms under a map with vertically moving dynamic obstacles. In Figure 9a, the path planned by ACO is almost attached to the obstacle, which lacks practical application value. In Figure 9c, it can be seen that the path obtained by APF is cluttered. The path obtained by the RRT shown in sub-figure rrtMap 3 is found to have a large angle occasionally, which will affect the tracking speed of the robot during the actual tracking process. The final path obtained by our proposed algorithm, shown in Figure 9d, maintains a certain distance from the obstacle, while maintaining a certain degree of smoothness.

### 4.3. Comparisons with Other Algorithms

This section mainly shows the comparison of the results of our method and comparison algorithms in batch simulations. The batch simulations in this paper refers to the average of 200 simulative results. We have shown the single-planning results of the comparison algorithm in Section 4.1 and Section 4.2. In order to make a more rigorous and scientific comparison of the proposed method and other comparison algorithms, we conducted batch simulations on each of the three maps and summarized the numerical results. Like most researchers, we chose path length and planning time as a measure of the superiority of the algorithm. Table 1 expresses the comparison results of the path lengths of the various algorithms. By comparison, it is found that, although the advantage is not particularly obvious, the proposed method has the shortest path on all maps. Table 2 exhibits the comparison results of planning time for all algorithms. And the BAS-APF method has obvious advantages in planning time.

From the above two tables, we can see that BAS-APF method has certain advantages in terms of path length and planning time, and the advantage in planning time is significant. In order to more intuitively demonstrate the advantages of the proposed method, we visualized the results.

**Figure 10:**
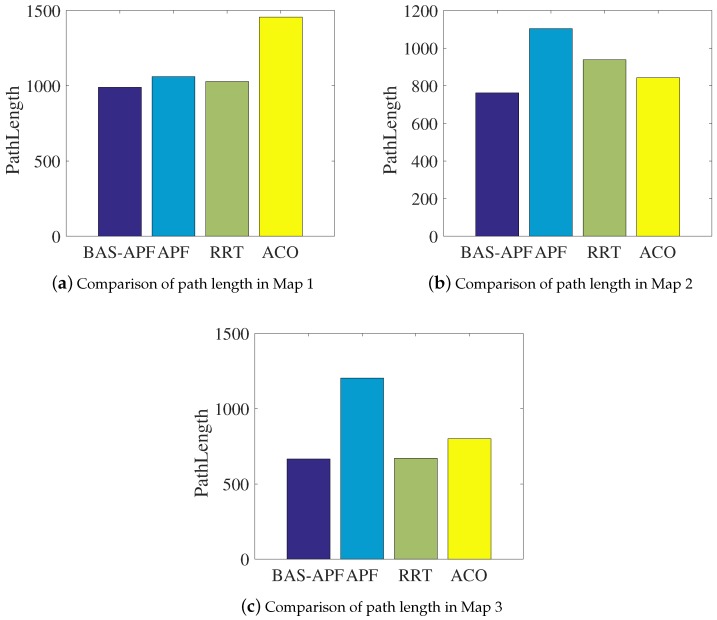
Comparison of path lengths on simulative maps.

**Figure 11:**
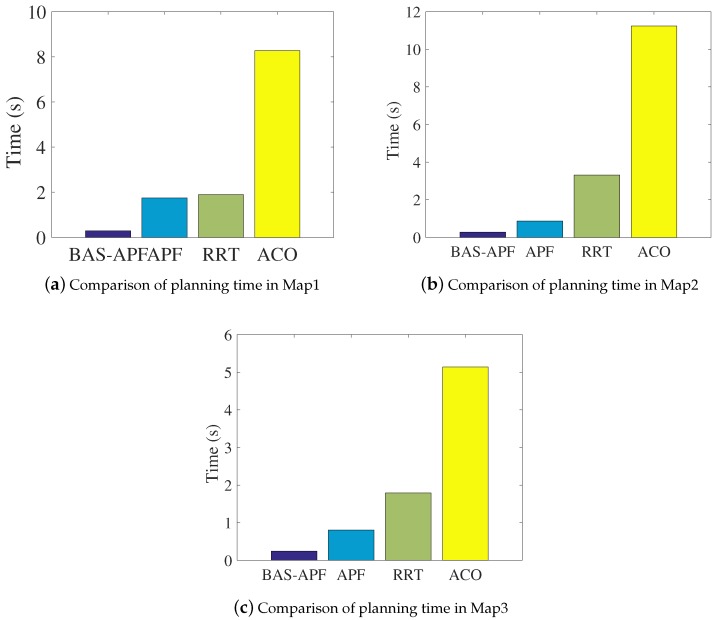
Comparison of planning time on simulative maps.

The results of the comparison are shown in detail in Figure 10 and Figure 11. Figure 10 is a comparison result of path lengths on respective maps. It can be seen that the performance of other algorithms is not stable, and the proposed method is more stable and optimal. Figure 11 displays the comparison of the planning time of each algorithm on the simulative map. The comparison of the three sub-figures in Figure 11 illustrates that our proposed method has significant advantages in planning time.

## 5. Conclusion and Future Work

This paper is concerned with the path planning problem of mobile robots in dynamic environments. We proposed a new two-stage hybrid method called BAS-APF to solve this problem. It could find a safe and feasible path, while ensuring real-time performance in a dynamic environment. Based on the real-time requirements of dynamic programming, the algorithm weighs path optimization and planning time, and it achieved good results in both aspects. In addition, we verified the effectiveness and robustness of the BAS-APF method through simulations. Moreover, the superiority of the proposed method was further verified by comparing it with several different types of classical path planning algorithms. The experiment test with real robots in real environments is not considered at the current state of this work. On the one hand, this is because the conditions of our laboratory are really limited, and the price of mobile robots is expensive, which we cannot afford, for the time being. On the other hand, the focus of this paper was mainly the research of algorithm, not the construction of the physical platform of mobile robot.

In the future, we intend to continue work on the following two aspects: One is that we would like to use real environment and real mobile robots for experiment tests in future work and realize the optimization on the accuracy of obstacle avoidance, so as to improve our algorithm and reflect the advantages of the proposed algorithm in the real environment. The other is to extend it to multi-objective planning so that it can simultaneously plan multiple mobile robots. 

## Figures and Tables

**Table 1:** Summary of comparison results of various algorithm path length.

	Algorithm	BAS-APF	APF	RRT	ACO
Map					
Map 1		**989.8**	1060.4	1028.6	1457
Map 2		**762.2**	1104.2	938.8	842.9
Map 3		**666.5**	1203	670.9	801.3

**Table 2:** Summary of comparison results of various algorithm planning time.

	Algorithm	BAS-APF	APF	RRT	ACO
Map					
Map 1		**0.287**	1.75	1.9	8.27
Map 2		**0.276**	0.871	3.31	11.24
Map 3		**0.244**	0.804	1.79	5.14
